# Identification of Residues in Lassa Virus Glycoprotein Subunit 2 That Are Critical for Protein Function

**DOI:** 10.3390/pathogens8010001

**Published:** 2018-12-26

**Authors:** Katherine A. Willard, Jacob T. Alston, Marissa Acciani, Melinda A. Brindley

**Affiliations:** 1 Department of Infectious Diseases, College of Veterinary Medicine, University of Georgia, Athens, GA 30602, USA; kwillard@uga.edu (K.A.W.); jacob.t.alston@gmail.com (J.T.A.); marissa.acciani@uga.edu (M.A.); 2 Department of Infectious Diseases, Department of Population Health, Center for Vaccines and Immunology, College of Veterinary Medicine, University of Georgia, Athens, GA 30602, USA

**Keywords:** Lassa virus, arenavirus, viral glycoprotein, viral entry, viral fusion, fusion protein

## Abstract

Lassa virus (LASV) is an Old World arenavirus, endemic to West Africa, capable of causing hemorrhagic fever. Currently, there are no approved vaccines or effective antivirals for LASV. However, thorough understanding of the LASV glycoprotein and entry into host cells could accelerate therapeutic design. LASV entry is a two-step process involving the viral glycoprotein (GP). First, the GP subunit 1 (GP1) binds to the cell surface receptor and the viral particle is engulfed into an endosome. Next, the drop in pH triggers GP rearrangements, which ultimately leads to the GP subunit 2 (GP2) forming a six-helix-bundle (6HB). The process of GP2 forming 6HB fuses the lysosomal membrane with the LASV envelope, allowing the LASV genome to enter the host cell. The aim of this study was to identify residues in GP2 that are crucial for LASV entry. To achieve this, we performed alanine scanning mutagenesis on GP2 residues. We tested these mutant GPs for efficient GP1-GP2 cleavage, cell-to-cell membrane fusion, and transduction into cells expressing α-dystroglycan and secondary LASV receptors. In total, we identified seven GP2 mutants that were cleaved efficiently but were unable to effectively transduce cells: GP-L280A, GP-L285A/I286A, GP-I323A, GP-L394A, GP-I403A, GP-L415A, and GP-R422A. Therefore, the data suggest these residues are critical for GP2 function in LASV entry.

## 1. Introduction

Mammalian arenaviruses are divided into two subgroups based on geographic distribution: Old World and New World [1]. Both subgroups contain human pathogens capable of causing severe hemorrhagic fever with high morbidity and mortality. Lassa virus (LASV), the pathogen that causes Lassa fever, is an Old World arenavirus endemic to West Africa. Each year, LASV infects several hundred-thousand people resulting in nearly 5000 deaths [2,3]. The 2018 outbreak in Nigeria was more extensive and had a higher case fatality rate (CFR) than normally recorded (CFR of confirmed cases was approximately 25% as of July 2018) [4], which exemplifies the need to develop LASV antivirals and vaccines. Human infections predominantly occur through zoonotic spread from the rodent host *Mastomys natalensis*, and potentially *Hylomyscus pamfi* and *Mastomys erythroleucus* [5,6]. Transmission can occur through direct contact with infected rodent hosts or exposure to rodent excreta/blood. In addition, person-to-person spread can occur through contact with infectious bodily fluids, putting healthcare workers at higher risk [7,8]. Due to the lack of vaccines and effective therapeutics, LASV is categorized as a class A pathogen [9].

The arenavirus particle consists of a host cell-derived lipid envelope encasing a bi-segmented RNA genome in an ambisense orientation. The envelope contains mature trimeric viral glycoprotein (GP) spikes that are responsible for attachment and entry into the host cell. The glycoprotein precursor (GPC) is produced as a type I membrane protein and is processed twice by host cell peptidases. First, a cellular peptidase in the endoplasmic reticulum (ER) cleaves the stable signal peptide (SSP) subunit from the precursor. Second, subtilisin kexin isozyme-1/site-1 protease (SKI-1/S1P) in the cis-Golgi cleaves GP1 from GP2 [10,11,12]. The arenavirus signal peptide is not degraded; instead, it becomes part of the trimeric glycoprotein complex serving as a chaperone assisting with protein processing, trafficking, and pH sensing [13,14,15,16]. The GP1 and GP2 subunits mediate receptor interactions and membrane fusion, respectively [17,18,19,20,21]. 

To enter the host cell, enveloped viruses must mediate fusion between the viral envelope and cellular membrane. The arenavirus glycoprotein contains two heptad repeat (HR) domains and an amino-terminal fusion peptide (N-FP) [22], characteristic of class I fusion proteins [19,20], and similar to those of retroviruses, filoviruses, paramyxoviruses, and influenza [17]. Unlike typical class I fusion proteins, the arenavirus GP2 also contains an internal fusion loop (I-FP), which helps mediate fusion [23,24]. Under low-pH, the arenavirus glycoprotein undergoes major conformational changes prior to initiating viral fusion [25]. Interaction between GP1 and lysosomal associated membrane protein 1 (LAMP1) dissociates GP1 from the trimer, activating the GP2 fusion protein [26,27]. During GP2 rearrangement, the fusion peptide/loop inserts into the host membrane. Once multiple GP2 subunits are triggered, the glycoproteins collapse into an energetically favorable conformation known as a six-helix bundle (6HB) [19]. Full collapse of the glycoprotein complex forms a fusion pore, which enables genome release into the cytoplasm [28]. 

The pre-fusion LASV GP and post-fusion GP2 of lymphocytic choriomeningitis virus (LCMV), a closely related Old World arenavirus, have been crystalized [19,29]. These two structures illustrate the major GP2 conformational changes that occur during fusion. Previous studies on arenavirus GP2 subunits have characterized both the C-terminal domain, required for interactions with SSP, and hydrophobic amino acids within the fusion peptide and fusion loop [23,30]. However, there has not been an extensive characterization of the GP2 subunit as a whole. Therefore, we produced a panel of GP mutants using either insertional or alanine-scanning mutagenesis and functionally characterized them to identify conserved residues that are critical for the fusion process. We identified several residues, that when changed to alanine, did not affect protein processing but inhibited GP2-mediated-fusion, suggesting these residues may be important for GP2 structural rearrangement or lipid interactions. 

## 2. Results

To identify critical residues in LASV GP2, we produced a panel of mutants. This panel included four hemagglutinin (HA) constructs in which the HA epitope tag was inserted at specific locations within GP2. It also included twenty-nine constructs in which conserved charged amino acids were changed to alanine; in two of these constructs, tandem charged residues were mutated together. Finally, we made twenty-six constructs in which hydrophobic amino acids were changed to alanine, which again included two constructs with tandem hydrophobic residues mutated in a single construct. 

### 2.1. Insertional Mutagenesis

**Figure 1:**
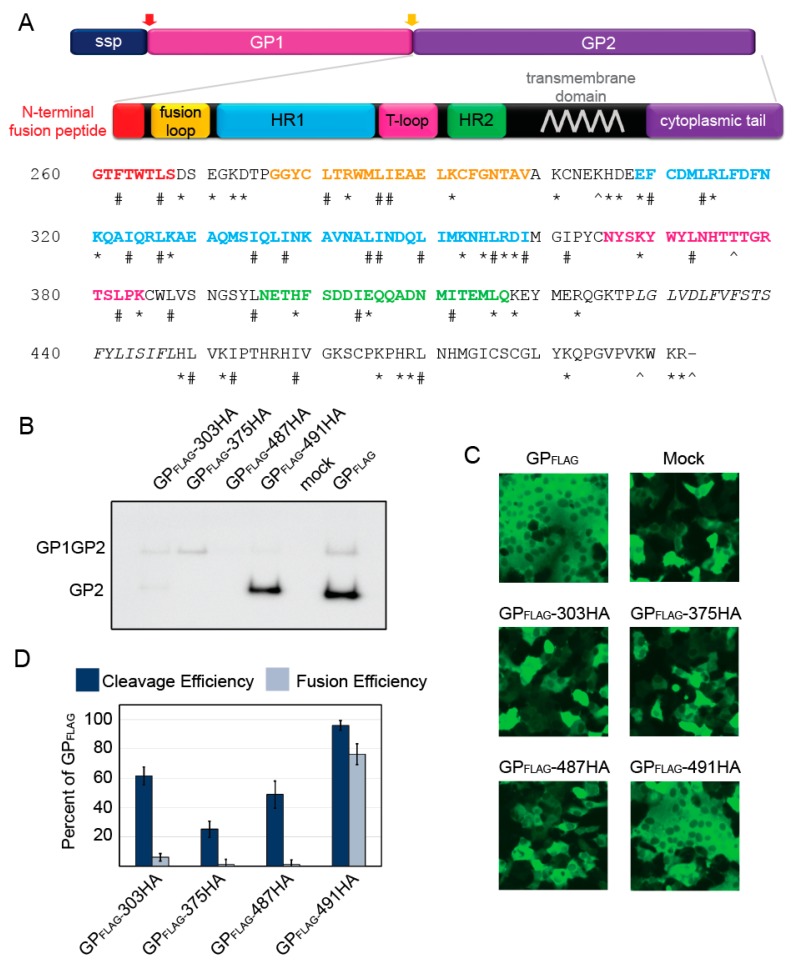
Functional analysis of LASV GP2 HA mutants. (**a**) Schematic of LASV GPC and amino acid sequence of GP2. The signal peptidase cleaves the SSP (red arrow) whereas SKI/S1P cleaves GP1-GP2 (yellow arrow). The known GP2 domains have been color coded, N-terminal fusion peptide (red); internal fusion loop (orange); heptad repeat 1 (blue); the T-loop (magenta); heptad repeat 2 (green); and the transmembrane domain is in italics (grey). The HA tags were inserted before the amino acids labeled with a ^, * denote charged, and # hydrophobic amino acids examined with alanine scanning. (**b**) Representative image of surface biotinylation to assess LASV glycoprotein processing to form GP2. GP1GP2 is the uncleaved glycoprotein precursor. LASV GP_FLAG_ was detected with an anti-FLAG antibody, M2, against the C-terminal GP2 3x FLAG tag. (**c**) Representative images of HA mutants in the cell-to-cell fusion assay. GP_FLAG_ is the parental LASV glycoprotein and mock represents cells transfected with only GFP (no glycoprotein). (**d**) LASV GP2 HA mutant cleavage and fusion efficiencies compared to parental GP_FLAG_. Error bars represent the standard error of the mean (SEM) from at least three independent trials.

The prefusion structure of LASV GP1-GP2 is compact, with the GP2 alpha helices stacked under the GP1 subunit [29]. Although the likelihood of inserting a peptide tag without disturbing the structure and function was low, we added HA peptides at two locations in the ectodomain. The first was inserted after position 303, which we predicted would fill the center core of the structure. The second was inserted after position 375, which added an HA peptide at the tip of the T-loop, close to the membrane in a surface exposed region (Figure 1A). We also added HA peptides to the cytoplasmic tail. One HA peptide was added at the C-terminus (residue 491), a position that is known to tolerate FLAG tag additions. An HA tag was also engineered after residue 487, to maintain the charged residues at the C-terminus. To characterize these HA mutants, we first assessed whether they were efficiently expressed on the cell surface and cleaved by SKI-1/S1P, releasing GP2. Only GP_FLAG_-491-HA was processed at levels comparable to parental GP_FLAG_ (Figure 1B,D). All of the remaining HA mutants had low (<50%) GP2 production levels compared to parental GP_FLAG_ (Table 1). Incubating cells expressing LASV GP with a low-pH buffer mimics the lysosomal low-pH environment and triggers GP fusion, resulting in robust syncytia formation [31]. Fusion efficiency is determined by comparing the extent of syncytia formation caused by the mutant GP to parental GP. Production of parental GP_FLAG_ protein induced extensive fusion that resulted in large syncytia after low-pH treatment. GP_FLAG_-491-HA was the only HA construct that induced fusion similarly to parental GP_FLAG_ (Figure 1C,D) as expected based on the surface levels. Taken together, these results suggest that the addition of the HA tag at these locations prevents efficient GP production and processing. 

### 2.2. Characterization of Charged Constructs

**Figure 2:**
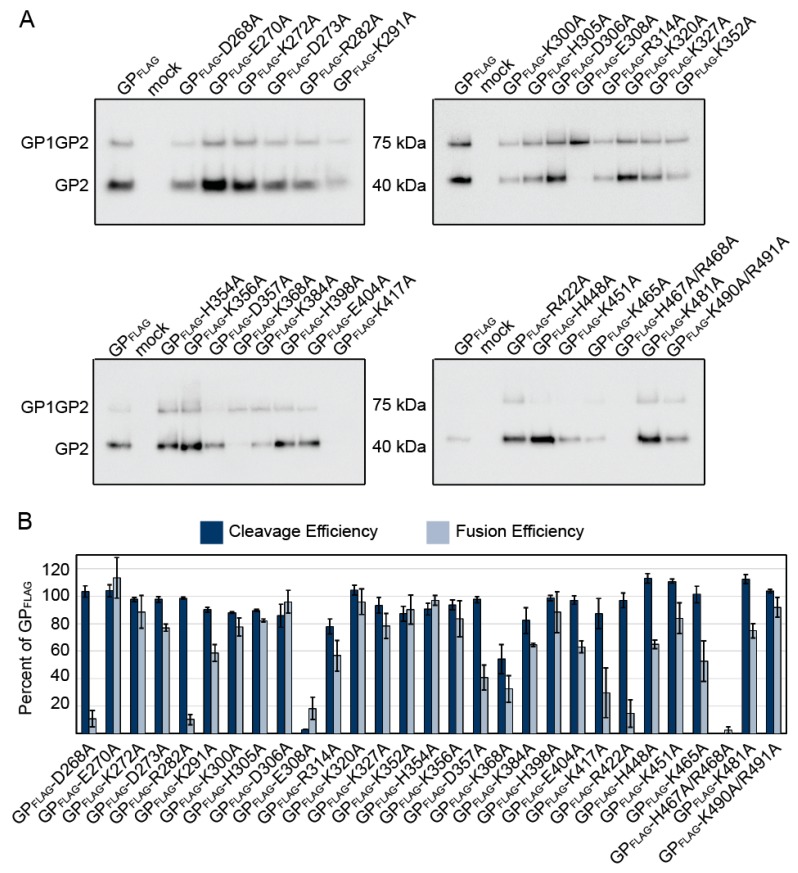
Functional analysis of charged residues in GP2. (**a**) Cell surface proteins were cross-linked to biotin and purified with streptavidin beads. Purified proteins were separated on SDS-PAGE and probed with an anti-FLAG antibody to detect GP, representative immunoblots are shown. (**b**) LASV GP2 mutant cleavage and fusion efficiencies compared to parental GP_FLAG_. Error bars represent the SEM from at least three independent trials.

In order to define individual residues important for GP2 refolding, we made more subtle mutations with alanine scanning mutagenesis. Since charged amino acids are important for protein organization and function [32], we began by mutating 29 highly conserved charged amino acid residues throughout the fusion-active subunit of LASV. We expressed the 29 constructs in Vero cells and monitored the levels of GP present on the cell surface to determine the production and cleavage efficiencies of the constructs (Figure 2A). All mutant glycoproteins except for GP-E308A and GP-H467A/R468A were cleaved producing GP2. GP-H467A/R468A did not produce detectable GP1GP2 or GP2, suggesting this mutation prevents proper protein folding and induced degradation. In contrast, GP-E308A produced a bright GP1GP2 band, which was not cleaved into GP2, indicating that GP1GP2 was produced and trafficked to the surface, but was not cleaved by SKI/S1P (Figure 2A, Table 2). 

To determine if the charged constructs produced functional GP, we assessed the fusion activity using the cell-to-cell based fusion assay. The majority of the mutant GPs produced syncytia at levels similar to parental GP (Figure 2B), suggesting the alanine substitutions at those positions did not impede interaction with the target membrane or low-pH induced protein conformational changes. Of the constructs that were efficiently processed into GP2, only three (GP-D268A, GP-R282A, and GP-R422A) reduced fusion (<20%) compared to parental LASV GP. D268 was adjacent to the N-terminal fusion peptide and R282 was within the internal fusion-loop, suggesting these charged residues were important for GP2 to effectively anchor into the target membrane. R422 was located between HR2 and the transmembrane domain, but was not resolved in either the pre-fusion or post-fusion structures. Alanine substitution at R422 retained GP1-GP2 processing and surface expression, but the decrease in cell-to-cell fusion suggests the arginine was important for the fusion process. 

### 2.3. Characterization of Hydrophobic Residues

**Figure 3:**
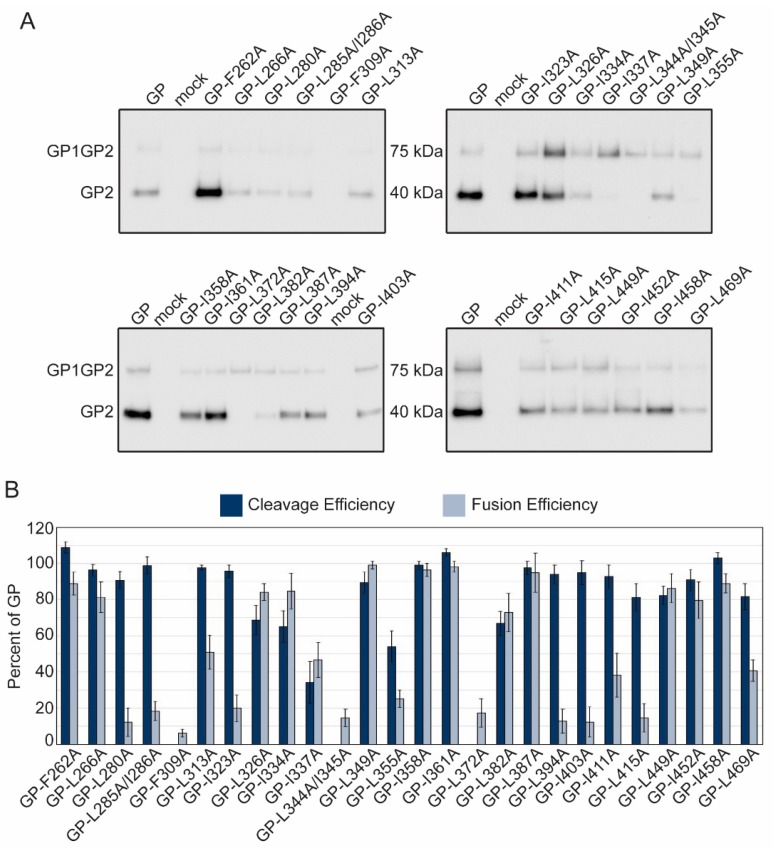
Functional analysis of conserved hydrophobic GP2 mutations. (**a**) Cell surface proteins were cross-linked to biotin and purified with streptavidin beads. Purified proteins were separated on SDS-PAGE and probed with anti-GP2 antibody (22.5D) representative immunoblots are shown. (**b**) LASV GP2 mutant cleavage and fusion efficiencies compared to parental GP. Error bars represent the SEM from at least three independent trials.

Hydrophobic residues within viral class I fusion proteins are involved in protein folding and are critical for the viral fusion peptides/loops to effectively insert into the target membrane [33]. Previous studies identified hydrophobic residues within the fusion domains and C-terminus of the LASV GP2 subunit that are required for fusion [23]. We built upon this work and characterized 26 mutations throughout the GP2 subunit to identify conserved, hydrophobic residues involved in GP structure and function. Once again, we analyzed cell surface expression and cleavage of the glycoprotein constructs by purifying proteins found on the surface of transfected cells. The majority of the mutated GP proteins were cleaved, but three constructs (GP-F309A, GP-L344A/I345A, and GP-L372A) did not produce detectible GP2 (Figure 3A, Table 2). GP-L344A/I345A and GP-L372A produced the GP1GP2 protein precursor indicating the mutation prevents recognition by SKI/S1P. GP-F309A was not detected in the surface material, indicating that the mutation is deleterious to GP precursor production or trafficking (Figure 3A). 

The cell-to-cell fusion assay results suggested many of the hydrophobic mutations reduced or eliminated syncytia formation. In total, there were six hydrophobic constructs, GP-L280A, GP-L285A/I286A, GP-I323A, GP-L394A, GP-I403A, and GP-L415A, that were efficiently cleaved (>80%) but fused <20% compared to parental LASV GP (Figure 3B). Constructs GP-L280A and GP-L285A/I286A reduced the hydrophobicity of the internal fusion loop, potentially preventing adequate insertion in the target membrane. I323 in HR1 and I403 and L415 in HR2 may have decreased efficient 6HB formation. L394 is a hydrophobic residue just outside of HR2 and may also inhibit 6HB formation. 

### 2.4. Transduction Efficiencies of Charged and Hydrophobic Mutants

Our cell-to-cell fusion assay examines GP2’s ability to undergo the low-pH induced conformational changes needed for fusion, but in an artificial system [34]. Lassa virus-to-cell fusion occurs in the lysosome, which contains different cellular proteins and lipids [35,36]. Therefore, to test whether the GP2 mutations affect viral entry, we pseudotyped our GP constructs onto vesicular stomatitis virus (VSV) particles lacking their native glycoprotein. VSV-LASV GP particles require LASV receptors for entry and trafficking to an endo-lysosomal compartment for efficient GP triggering. We used HAP1 and HAP1-ΔDAG haploid cell lines in our transduction assays to examine different entry mechanisms. Both cell lines have been extensively characterized for LASV entry [18,37]. HAP1 cells express the primary LASV receptor, α-dystroglycan (α-DG), and LASV entry occurs most efficiently when this receptor is present. HAP1-ΔDAG cells lack α-DG but contain secondary receptors that enable LASV entry through less efficient routes [18,31].

The LASV glycoprotein precursor must be cleaved into GP1 and GP2 to induce fusion. Therefore, we examined transduction efficiency for two sets of constructs: parental-like (constructs that produced over 80% cleaved GP2 and over 50% fusion efficiency compared to parental GP) and fusion-defective (constructs that produced over 80% cleaved GP2 but had less than 20% fusion efficiency compared to parental GP). We hypothesized that the parental-like mutants should be able to effectively transduce these cells whereas the fusion-defective mutants would have comparatively low transduction efficiencies. Because GP2 is not directly involved in receptor interactions, we expected few differences in the transduction efficiencies between HAP1 and HAP1-ΔDAG cell lines. 

**Figure 4:**
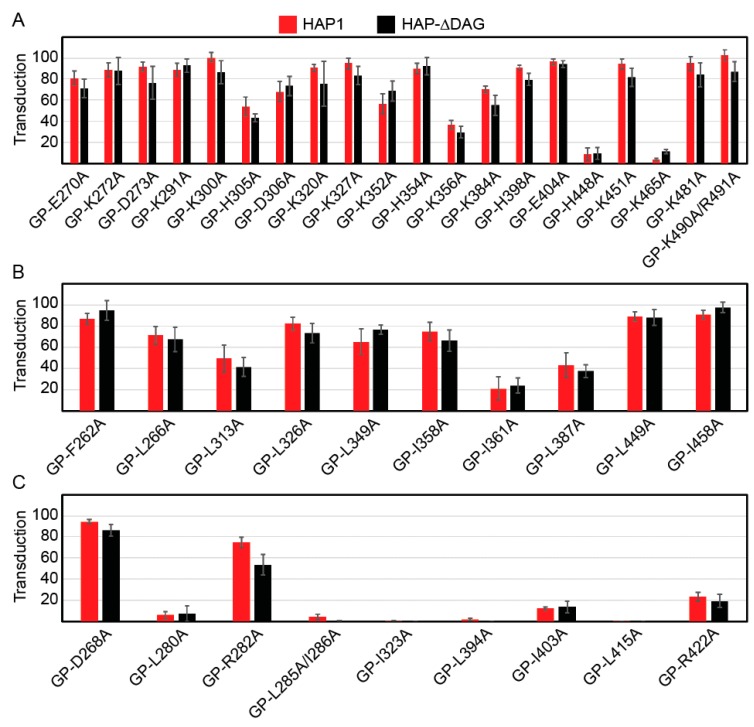
Transduction efficiencies of parental-like charged (**a**), parental-like hydrophobic (**b**), and fusion-defective (**c**) GP2 mutants. HAP1 and HAP1-ΔDAG1 cells were transduced with VSVΔG-LASV GP constructs encoding GFP. Transduction was quantified using flow cytometry by gating for GFP-positive cells. Transduction efficiency for each construct was normalized to parental LASV GP particle transduction for each cell line. All data are based on the average and standard error of the mean of at least three replicate experiments.

We examined the transduction efficiencies of 20 charged (Figure 4A, Table 2) and 10 hydrophobic parental-like constructs (Figure 4B, Table 2). The majority of the charged mutant constructs transduced cells relative to the level of GP produced in the cells. However, three constructs, GP-H305A, GP-K356A, and GP-K465A produced near wild-type levels of cleaved GP, but transduced poorly, suggesting these residues may be important in particle incorporation or fusion activity in the lysosome. Surprisingly the GP-H448A construct was unable to transduce either HAP cell line despite high cell surface production. The majority of hydrophobic constructs also transduced cells at rates similar to the levels of cleaved GP (Figure 4B). Only GP-I361A, a residue adjacent to HR-N, was cleaved and fused at parental GP levels but inefficiently transduced cells. As expected, construct transduction efficiencies did not significantly differ between cell lines, confirming that the GP2 mutations are not altering receptor interactions.

While we expected poorly-fusing mutant GPs to demonstrate equally reduced transduction, two mutant GPs, GP-D268A and GP-R282A, transduced cells efficiently (>80% and >50% respectively) (Figure 4C, Table 2). Both of these charged residues are part of the fusion peptide region; D268 is adjacent to the N-FP and R282 is within the fusion loop. The lipid and protein content of the plasma membrane and lysosomal membrane are distinct [36]. Thus, the removal of the charged residue may have altered the low-pH induced conformation of the fusion peptide, preventing proper insertion at the plasma membrane, but retaining activity in the lysosomal membrane. 

## 3. Discussion

**Figure 5:**
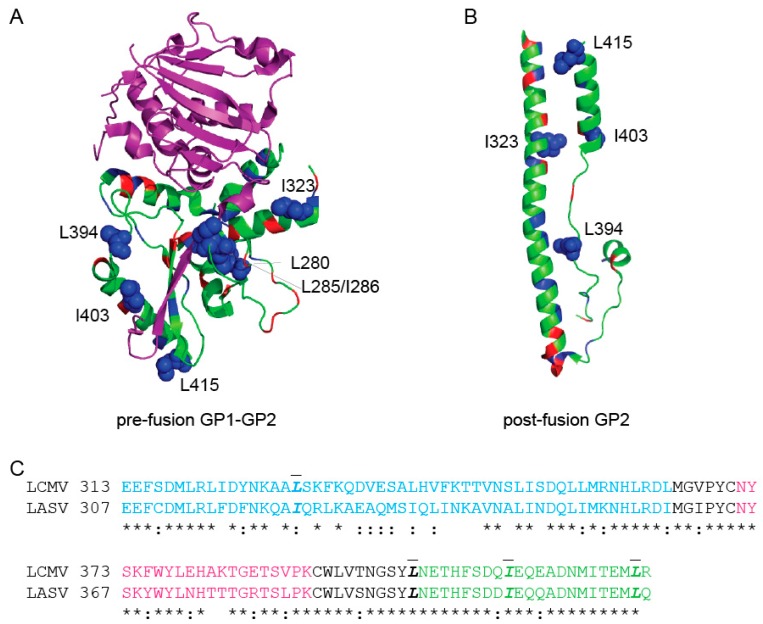
Mapping the fusion-defective charged and hydrophobic mutations on pre-fusion LASV GP2 and post-fusion LCMV homology model. (**a**) The LASV GP1-GP2 monomeric pre-fusion crystal structure (PDB 5vk2) [29] The GP1 subunit is shown in purple and the GP2 subunit is shown in green. The residues targeted in this study are highlighted, charged residues (red) and hydrophobic (blue). Residues found to be critical for GP2 fusion activity are shown in spheres (L280, L285/I286, I323, L394, I403, and L415) (**b**) The LCMV (an Old World arenavirus closely-related to LASV) GP2 post-fusion crystal structure (PDB 3mko) [19]. Homologous residues are highlighted as in part (a). (**c**) Amino acid alignment of LCMV GP2 region crystallized with corresponding region of LASV GP2. Identical residues are indicated with a (*), whereas conservative replacements are indicated by (:). Residues labeled in the structure are in bold-italics and contain a dash above the residues. All structures were rendered with PyMol.

In this study, we produced and characterized a library of 59 LASV GP2 mutants to identify residues involved in GP2 function. We identified 14 residues (E308, F309, I334, I337, L344/I345, L355, K368, L372, L382, K417, H467/R468, and L469) that are critical for GP folding, trafficking, or SKI/S1P recognition, evidenced by the lack of GP2 present in cell surface material. Twenty-one charged or hydrophobic residues could be changed to alanine without significantly altering protein production, localization or function, suggesting that GP2 can tolerate mutations at those locations. In total, nine constructs were efficiently cleaved (at least 80% of parental GP), yet failed to produce syncytia at levels similar to parental GP. Many of these residues, including D268, L280, R282, and L285/I286, are part of the fusion peptide domain. Both L280 and L285-I286 within I-FP impaired both cell-to-cell and virus-to-cell fusion, suggesting these hydrophobic residues may be critical for I-FP insertion, as expected (Figure 5). Surprisingly, GP-D268A and GP-R282A mutant proteins failed to induce cell-to-cell fusion, but were efficient in virus-to-cell fusion when incorporated onto VSV particles. These data suggest the charged residues are required for mediating fusion at the plasma membrane that is predominantly saturated lipids and sterols, but are less important when fusing in the lysosomal membrane, which contains low levels of cholesterol [36,38]. Similar lipid-dependent fusion occurs with other viruses; efficient insertion of the Dengue fusion peptide requires specific lipids within the late endosomes and fails to mediate low-pH fusion at the plasma membrane [39].

While many of the residues that impacted fusion localized to the fusion peptide, five residues were part of other GP2 domains (Figure 5). I323 is part of the HR1 domain, I403 and L415 are in the HR2 domain, and L394 is the amino acid preceding HR2 (Figure 5). These residues may be critical for 6HB formation [19]. Residue R422 falls right next to the membrane but was not resolved in either crystal structure. Removal of the charged residue may impact the final 6HB structure and inhibit fusion. 

Of the seven constructs that produced cleaved GP2 but failed to transduce cells (GP-L280A, GP-L285A/I286A, GP-I323A, GP-L394A, GP-I403A, GP-L415A, and GP-R422A), six were hydrophobic residues. While all of the constructs retained high cleavage efficiencies, several (GP-L280A, GP-L285A/I286A, GP-L394A, GP-I403A, and GP-L415A) produced decreased levels of GP on the surface, which may contribute to the decreased transduction. 

Three of our hydrophobic constructs, GP-F262A, GP-L266A, and GP-L280A were previously characterized [23]. Klewitz et al. found GP-F262A and GP-L266A produced very little protein on the surface and had no fusion activity [23]. However, our cleavage data suggests that both GP-F262A and GP-L266A are surface-expressed near or above parental GP levels and induced both cell-to-cell and virus-to-cell fusion (Figure 3A). These phenotypic differences may be due to a difference in our transfection protocols, harvesting cells at 36 h versus 24 h, which allows more time for GP to traffic to the cell surface. We both found GP-L280A produced cleaved GP2, albeit at a decreased level, but was unable to induce fusion. Therefore, while the hydrophobic nature of the N-FP and I-FP is important, some individual residues can be made less hydrophobic while preserving functionality. Both data sets agree that the internal fusion peptide region is important for protein function.

Taken together, these data demonstrate that both conserved hydrophobic and charged residues throughout GP2 are required for optimal protein function. Specifically, the data highlighted specific residues near or within the I-FP, HR1, and HR2 domains that play critical roles in fusion. 

## 4. Materials and Methods 

Cell Lines and Transfections 

Vero cells stably expressing human SLAM were maintained in Dulbecco−s modified Eagle−s medium (DMEM) with 5% (*v*/*v*) fetal bovine serum (FBS) and incubated at 37 °C and 5% CO_2_ [40]. HAP1 and HAP1-ΔDAG1 cells (Horizon Discovery, Cambridge, UK) were maintained in Iscove’s media supplemented with 10% (*v*/*v*) FBS and incubated at 37 °C and 5% CO_2_. All transfections were performed with GeneJuice (Millipore, Burlington, MA) according to the manufacturer’s instructions.

Expression Vectors and Mutagenesis 

The LASV GPC protein coding sequence was codon optimized for mammalian expression and cloned into a pcDNA3.1 intron vector. A CMV promoter initiated gene expression, and we included a β-globin intron in the 5’ untranslated region (UTR) to increase protein production [31]. We added a carboxy-terminal 3xFLAG tag to the GP2 cytoplasmic tail to biochemically detect HA and charged constructs. HA insertions and point mutations were created with QuikChange mutagenesis and PfuTurbo-HS polymerase (Agilent, Santa Clara, CA). We verified the presence of each mutation with DNA sequence analysis, and a complete sequence information is available upon request.

Surface Biotinylation

Vero cells were transfected (as described above) with plasmid DNA encoding the indicated LASV GPC construct. Thirty-six hours following transfection, cells were washed with cold PBS and incubated with 0.5 mg/mL sulfosuccinimidyl-2-(biotinamido) ethyl-1,3-dithiopropionate (ThermoFisher, Waltham, MA) for 30 min on ice to tag cell surface proteins with biotin [41]. The reaction was quenched with Tris-HCl, and cells were lysed in M2 lysis buffer (50 mM Tris, pH 7.4, 150 mM NaCl, 1 mM EDTA, 1% Triton X-100) at 4°C, then centrifuged (20,000× g, 15 min, 4°C). The clarified lysate was rotated with streptavidin sepharose beads (GE Healthcare, Chicago, IL) for 60 min. Following incubation, the streptavidin sepharose beads were washed in buffer 1 (100 mM Tris, 500 mM lithium chloride, 0.1% Triton X-100) and then in buffer 2 (20 mM HEPES (pH 7.2), 2 mM EGTA, 10 mM magnesium chloride, 0.1% Triton X-100). The samples were then incubated in urea buffer (200 mM Tris, pH 6.8, 8 M urea, 5% sodium dodecyl sulfate (SDS), 0.1 mM EDTA, 0.03% bromophenol blue, 1.5% dithiothreitol) for 30 min at 55°C and analyzed using an immunoblot.

Antibodies and Immunoblots 

After surface biotinylation, samples were separated by gel electrophoresis on 4-20% Nu-PAGE gels (ThermoFisher, Waltham, MA) and transferred to polyvinylidene difluoride (PVDF) membranes (GE Healthcare). HA and charged GP constructs were detected with an antibody against the Flag epitope tag (M2; Sigma, Burlington, MA) and a mouse IgG horseradish peroxidase (HRP)-conjugated secondary antibody (Jackson ImmunoResearch, West, Grove, PA). Hydrophobic constructs were detected with an antibody against LASV GP2 (22.5D), kindly provided by Dr. James Robinson (Tulane University), and a human IgG HRP-conjugated secondary antibody (Jackson). Immunoblots were visualized with SuperSignal West Dura Extended Duration Substrate (ThermoFisher, Waltham, MA) and a ChemiDoc digital imaging system (Bio-Rad, Hercules, CA). Immunoblots were quantified using ImageLab software (Bio-Rad, Hercules, CA). 

Cell-to-Cell Fusion Assay 

Vero cells were co-transfected with LASV GP mutants and pmaxGFP (4:1 ratio). Forty hours following transfection, media was removed and replaced with PBS (pH 4) and incubated (37 °C and 5% CO_2_) for 30 min to allow glycoprotein triggering. The PBS was replaced with warm DMEM and cells were incubated for an additional 3 h to enable membrane rearrangement and syncytia formation. Four representative pictures of the fusion were taken using Zoe microscope (Bio-Rad) (20× magnification) and unfused cells were counted. Fusion efficiency was quantified using the following equation:

$$Fusion\;=\;\frac{\left(unfused\;cells\;in\;GFP\;transfected-unfused\;cells\;in\;mutant\;transfected\right)}{\left(unfused\;cells\;in\;GFP\;transfected-unfused\;cells\;in\;parental\;GPC\;transfected\right)}\times 100$$

Each mutant was assessed for fusion in at least three independent experiments.

VSV Pseudoparticle Production and Transductions

GP constructs lacking the C-terminal 3xFlag tag were used to make vesicular stomatitis virus (VSV) pseudotyped particles. Vero cells were transfected with LASV GP DNA. Thirty-six hours following transfection the cells were transduced with VSVΔG-GFP particles pseudotyped with VSV-G (MOI 1) for one hour (courtesy of Dr. Michael Whitt; KeraFAST, Boston, MA) [42]. The particle-containing media was then replaced with fresh DMEM. VSVΔG-GFP particles displaying the LASV GP were collected 8 h following the transduction. These particles were applied onto HAP1 and HAP1-ΔDAG1 cells. A larger volume of particles (4 times as much) was used to transduce HAP1-ΔDAG1 cells to overcome the decreased transduction efficiency when cells are missing the primary α-DG receptor [18]. The number of GFP positive cells was enumerated in a flow cytometer. Results are displayed as the percent of GFP positive cells present in a population of 10,000 live cell events compared to parental GP transduction.

## Figures and Tables

**Table 1:** Summary of Fusion and GP cleavage of HA mutants.

Mutant	GP2 Protein Expression ^1^	Cleavage Efficiency ^1^	Fusion Activity ^1^
303-HA	14.3 ± 3.5	62.8 ± 6.1	6 ± 2.6
375-HA	1.1 ± 0.1	25.4 ± 5.6	0 ± 3.6
487-HA	1.6 ± 0.4	50.2 ± 9.1	0 ± 3.2
491-HA	55.0 ± 16.1	98.5 ± 3.5	76.3 ± 7.1

^1^ All values are displayed as percentage of GP_FLAG_ control ± SEM.

**Table 2:** Summary of GP2 Expression, GP Cleavage, Cell-to-cell Fusion, and Transduction Data.

Mutant ^1^	Mutant Type ^2^	GP2 Protein Expression ^3^	Cleavage Efficiency ^3^	Fusion Activity ^3^	Transduction Efficiency ^3^	
					HAP1	HAP1-ΔDAG
F262A	H	198.3 ± 62.5	108.8 ± 3.1	88.9 ± 6.4	87.2 ± 5.2	94.7 ± 9.3
L266A	H	56.2 ± 6.7	96.3 ± 3.2	81.1 ± 8.4	71.5 ± 8.5	67.7 ± 11.4
***D268A***	***C***	***118.6 ± 46.2***	***103.4 ± 4.2***	***10.8 ± 5.0***	***94.1 ± 1.8***	***86.0 ± 5.5***
E270A	C	187.4 ± 41	104.1 ± 4.2	113.3 ±14.8	80.7 ± 6.7	71.1 ± 8.9
K272A	C	136.2 ± 17.3	97.7 ± 1.5	88.5 ± 11.8	88.5 ± 6.5	87.6 ± 13.2
D273A	C	101.4 ± 12.8	97.7 ± 2	77.1 ± 2.7	91.5 ± 4.2	76.2 ± 15.6
***L280A***	***H***	***45 ± 12.2***	***90.7 ± 4.7***	***12.1 ± 7.9***	***6.1 ± 3.3***	***7.6 ± 7.1***
***R282A***	***C***	***80.7 ± 11.2***	***98.6 ± 0.7***	***10.1 ± 3.6***	***74.2 ± 5***	***53.5 ± 9.6***
***L285A/I286A***	***H***	***63.7 ± 13.5***	***98.9 ± 4.7***	***18.4 ± 5.4***	***4.2 ± 2.6***	***0.5 ± 0.5***
K291A	C	43.4 ± 15.3	90.2 ± 1.8	58.5 ± 16.2	88.5 ± 6	92.8 ± 6.8
K300A	C	49 ± 16.5	88.1 ± 0.6	77.6 ± 6.5	100.8 ± 4.9	86.4 ± 10.6
H305A	C	95.8 ± 34.7	89.6 ± 0.7	82.2 ± 1.2	53.8 ± 8.8	43.2 ± 3.9
D306A	C	98.5 ± 31.1	85.8 ± 8.3	96 ± 8.1	67.8 ± 9.6	73.2 ± 8.9
E308A	C	0 ± 0	3.0 ± 1.0	18.2 ± 8.3		
F309A	H	0 ± 0	0 ± 0	6.2 ± 2.1		
L313A	H	63.9 ± 10	97.6 ± 1.4	50.8 ± 9.3	49.5 ± 12.8	41.5 ± 8.8
R314A	C	35.7 ± 11.8	78.1 ± 5.4	56.8 ± 11.2		
K320A	C	118.6 ± 27.3	104.4 ± 3.5	96 ± 9.1	90.4 ± 3.4	75.3 ± 21
***I323A***	***H***	***116.4 ± 26.2***	***95.6 ± 3.5***	***20 ± 7.3***	***0.5 ± 0.3***	***0 ± 0***
L326A	H	57.3 ± 13.3	68.6 ± 8.1	84.1 ± 4.6	82.6 ± 6	73.6 ± 9.4
K327A	C	69.8 ± 12.8	93.4 ± 5.6	78.3 ± 9	95.0 ± 5.5	83.1 ± 8.7
I334A	H	18.8 ± 6.6	65 ± 8.8	84.7 ± 10		
I337A	H	12.2 ± 4.9	34.2 ± 11.5	46.5 ± 9.6		
L344A/I345A	H	0 ± 0	0 ± 0	14.7 ± 4.8		
L349A	H	54.7 ± 15.1	89.3 ± 5.8	99.1 ± 2.2	64.4 ± 12.2	76.9 ± 4.6
K352A	C	32.1 ± 8.2	87.3 ± 5.3	90.3 ± 10.7	56.5 ± 9.5	68.5 ± 9.5
H354A	C	79.9 ± 26	90.8 ± 4.3	96.9 ± 3.5	89.7 ± 4.9	92.2 ± 8.7
L355A	H	14.8 ± 4.1	53.9 ± 8.7	25.2 ± 4.9		
K356A	C	85.7 ± 37.1	93.6 ± 3.6	83.5 ± 13.3	36.8 ± 4.0	29.4 ± 5.5
D357A	C	39.4 ± 13.3	97.6 ± 2.2	40.6 ± 9.1		
I358A	H	68.2 ± 11.7	99 ± 2.3	96.3 ± 3.7	75.1 ± 8.5	66.7 ± 9.9
I361A	H	117.5 ± 28.8	106 ± 2.1	98.1 ± 3.1	21.3 ± 11	24.1 ± 7.2
K368A	C	13.6 ± 2.8	54.3 ± 10.7	32.5 ± 9.6		
L372A	H	0 ± 0	0 ± 0	17.3 ± 7.8		
L382A	H	25.6 ± 8.9	66.8 ± 6.5	72.9 ± 10.5		
K384A	C	24.2 ± 3.3	82.3 ± 9.2	64.6 ± 1.4	70.1 ± 3.3	55.1 ± 9.4
L387A	H	71.4 ± 16.4	97.4 ± 3.9	94.8 ± 10.8	43.3 ± 11.7	37.6 ± 5.9
***L394A***	***H***	***45.7 ± 4.9***	***93.8 ± 5.3***	***12.8 ± 6.7***	***2 ± 1***	***0 ± 0***
H398A	C	165 ± 93.7	98.7 ± 1.8	88.3 ± 14.8	90.5 ± 2.0	79.3 ± 5.6
***I403A***	***H***	***57.8 ± 13.7***	***94.9 ± 6.7***	***12.3 ± 8.2***	***12.4 ± 1.4***	***13.8 ± 5.4***
E404A	C	43.5 ± 19.8	97 ± 3	63.1 ± 4.3	96.3 ± 3.3	93.9 ± 2.9
I411A	H	63.7 ± 20.1	92.7 ± 6.4	38.1 ± 11.9		
***L415A***	***H***	***34.1 ± 10.7***	***81.3 ± 7.5***	***14.6 ± 7.8***	***0.3 ± 0.2***	***0 ± 0***
K417A	C	22.1 ± 12.4	87.3 ± 11	29.5 ± 18		
***R422A***	***C***	***286.2 ± 138.8***	***97 ± 5.5***	***14.6 ± 10.1***	***23.4 ± 4.1***	***19.2 ± 6.2***
H448A	C	560.5 ± 235.9	113 ± 3.7	64.9 ± 3.1	8.9 ± 5.6	9.8 ± 5.5
L449A	H	41.6 ± 16.9	82.2 ± 5	86.2 ± 8	89.3 ± 4.3	88.2 ± 7.4
K451A	C	225.4 ± 48.5	110.9 ± 1.6	84.1 ± 11.2	94.4 ± 4.9	81.3 ± 8.8
I452A	H	40.1 ± 13.5	90.8 ± 5.9	79.5 ± 10.1		
I458A	H	95.7 ± 26.7	103 ± 3.1	88.9 ± 5.2	90.9 ± 4.2	97.9 ± 5.0
K465A	C	91.6 ± 28.9	101.4 ± 6	52.7 ± 14.8	3.8 ± 1.3	11.3 ± 2.1
H467A/R468A	C	0 ± 0	0 ± 0	2.4 ± 2.4		
L469A	H	19.2 ± 3.1	81.7 ± 7.2	40.6 ± 5.9		
K481A	C	393.2 ± 200.5	112.3 ± 3.3	74.9 ± 5.2	94.9 ± 6.7	83.8 ± 11.5
K490A/R491A	C	223.7 ± 64.1	103.6 ± 1.2	91.8 ± 7.1	102.8 ± 5.1	86.8 ± 9.3

^1^ Mutations that impaired GP2 function (>80% cleavage efficiency and <20% fusion activity) are bold and italicized. ^2^ Table 1 abbreviations. H: Hydrophobic; C: Charged. ^3^ All values are displayed as a percentage of GP control ± SEM.
